# Determination of Kaurenoic Acid in *Acanthopanax trifoliatus* by Ultra-High Performance Liquid Chromatography Coupled with Tandem Mass Spectrometry (UHPLC-MS/MS)

**DOI:** 10.1038/s41598-020-60426-3

**Published:** 2020-02-25

**Authors:** Qun Peng, Jianyuan Chen, Hanying Duan, Chao Wang

**Affiliations:** 1 Department of Food Science and Technology, Jinan University, Guangzhou, 510632 China; 2 Division of Research and Development, Kingmed Diagnostics, Guangzhou, 510330 China

**Keywords:** Secondary metabolism, Secondary metabolism, Secondary metabolism, Secondary metabolism, Predictive markers

## Abstract

*Acanthopanax trifoliatus* (L.) Merr. (*A. trifoliatus)* belongs to the family *Araliaceae*, which is called “Le Cai”, and is an indigenous plant to Guangdong Province that has been prevalently planted for years. *A. trifoliatus* is used in folk medicine and has ginseng-like activity. Kaurenoic acid ((**−**)-kaur-16-en-19-oic acid, KA) is a kaurane-type diterpenoid that is regarded as a major compound in *A. trifoliatus*. Early studies have reported the determination of KA by HPLC capillary electrophoresis. However, KA could not be completely separated from other components in the plant extract by HPLC because of their similar molecular structures and physical and chemical properties. UHPLC-MS/MS could be a useful tool to identify and quantify KA. In the present work, a UHPLC-ESI-MS/MS method for determining KA in *A. trifoliatus* was developed and validated. KA was extracted from lyophilized *A. trifoliatus* leaves by ultrasound-assisted extraction and further purified by solid phase extraction (SPE). KA was quantified and separated on an Accucore C_18_ LC column. Mass spectrometry with multi-reaction monitoring (MRM) and quantitative fragment ion/product ion (*m/z*: 301.3/301.3) in ESI negative mode was used for quantification. The intra-assay and inter-assay relative standard deviation (R.S.D.) were 2.8% and 3.2%, respectively. The inter-person R.S.D. on the same day was 3.6%. The inter-instrument R.S.D. with the same model on the same day was 2.9%. The recoveries evaluated upon spiking three different concentrations of KA were above 97%. A minor matrix effect of 94% was observed. This method has been applied successfully for the determination of KA in *A. trifoliatus* leaves.

## Introduction

*Acanthopanax* is a plant genus that embraces 18 plant species that are mainly distributed in Asia and the far-eastern region of Russia^1,2^. One of the *Acanthopanax* species is *A. trifoliatus*, which is called “Le Cai” in Chinese, and is an indigenous plant in Enping, Guangdong Province that has been prevalently planted for years. The planting area has reached 420 hectares, and the total value is worth approximately 120 million. *A. trifoliatus*, with ginseng-like activity, has been used in folk medicine in southern China since old time to treat sinew, bone pains, rheumatism, bruises, neuralgia, impotence, gout, hepatitis and diabetes^3–5^. In Cambodia, Laos, and Vietnam, *A. trifoliatus* has been used to treat nervous affections and improve memory^6,7^. It has also been shown to have a good curative effect on the common cold, jaundice, gastric pain, diarrhea and ulcers^8^. A decoction of leaves, young shoots or the bark of *A. trifoliatus* has been used to treat tuberculosis and lung hemorrhages^9^. Some studies have indicated that extracts of *A. trifoliatus* possess several beneficial biological effects, including anti-inflammatory, immunostimulatory, and antioxidant properties, protein tyrosine phosphatase inhibitory activity and cytotoxic activities towards some types of cancer cells^10–12^.

Moreover, the young leaves and shoots of *A. trifoliatus* are popularly consumed as vegetables in traditional southern Chinese cuisine that can be stir-fried or cooked in a soup^13^. In addition, the local people of southern China have been using the leaves of this plant to make tea for daily consumption for at least 10 years for daily health management. This plant is also an ingredient in ‘leicha’, a traditional Chinese herbal tea that is believed to have a powerful tonic effect^14^. *A. trifoliatus* is usually planted from March to October and is a rigid shrub 1 ± 0.4 m high. It is spiny with alternate palmate leaves and toothed margins, and it has a typical crisp flavor with some bitterness^15^.

**Figure 1:**
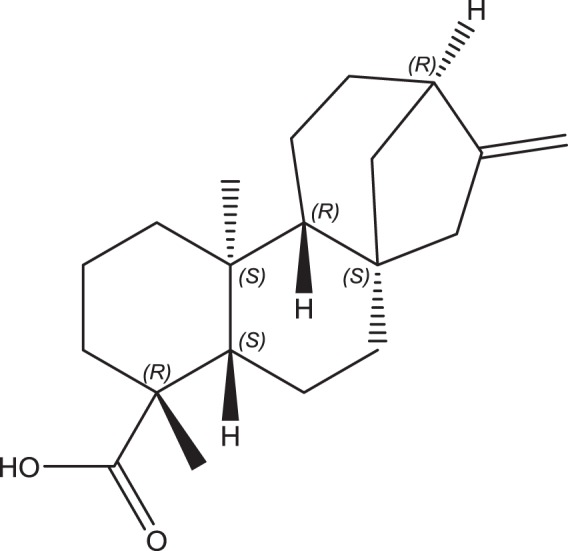
Chemical structure of KA.

The leaves of this plant contain a diverse range of components, including diterpenoids, lignans, triterpenoids, polyacetylenes, phenylpropanoids, and flavonoids^16,17^. Of these components, KA has been reported as an important ingredient in *A. trifoliatus* for its potent biological activities^11,18–20^. KA is a kaurane-type diterpenoid and is regarded as a key intermediate in the biogenesis of gibberellins and other phytohormones that regulate the growth and development of higher plants and some fungal metabolites^21,22^. The structure of KA is shown in Fig. 1.

Therefore, for the effective utilization and quality control of natural resources, analysis of KA in *A. trifoliatus* could be very important. Several reports have determined KA contents by HPLC or GC. Early studies found that KA could not be completely separated from other components in plant extracts by HPLC because of its similar molecular structure and physical and chemical properties^4,23,24^.

UHPLC-MS/MS could be a useful tool to identify and quantify KA. In this study, a UHPLC-MS/MS method was developed for the quantitative determination of KA in the leaves of *A. trifoliatus* to use the leaves as an effective medical resource. Although the determination of KA by LC-MS/MS had been developed in early reports, this is the first fully validated method in terms of extraction, UHPLC-MS/MS conditions, linearity, precision, accuracy, limit of detection (LOD) and limit of quantitation (LOQ).

## Results and Discussion

### Effect of sample preparation on KA recovery

To investigate the influence on ultrasound time on the recovery of KA, 0.1 g of *A. trifoliatus* leaves were extracted in 100 mL of 100% methanol by ultrasonic extraction for 15, 30 or 60 min, and the determined concentrations were 4.1 ± 0.3, 4.2 ± 0.3 and 4.2 ± 0.0 μg/mg solid, respectively. From these results, increasing the ultrasound treatment time slightly increased the extracted components but there was no significant difference after applying the ANOVA *post-hoc* test. Additionally, the R.S.D. of these three treatment times was 2.8–3.4%. Therefore, a 15 min ultrasound time was chosen for further analysis.

Different sample extraction amounts were also optimized, including 0.1, 0.2, 0.3 and 0.5 g, and the corresponding results were 4.0 ± 0.8, 4.1 ± 0.0, 4.2 ± 0.1 and 4.1 ± 0.1 μg/mg solid, respectively. The content of KA by weighting different sample extraction amounts were shown in Supplement 1. The R.S.D. of the four different sample extraction amounts was 2.3%. By the ANOVA *post-hoc* test, there was no significant difference between the four exaction amounts. Therefore, to decrease the matrix effect, 0.1 g of sample was chosen in this study.

### Optimization of UHPLC conditions on KA recovery

In this study, UHPLC conditions were mainly optimized in terms of the type of column, column temperature, flow rate and solvent effect. Two different UHPLC columns were tested with regards to method reproducibility, including an Accucore C_18_ UHPLC column (100 × 2.1 mm, 2.6 μm, Thermo Scientific, USA) and a Zorbax Eclipse Plus C_18_ column (2.1 × 50 mm, 1.8 μm, Agilent Technologies, USA). The retention times of KA on the Accucore and Zorbax columns were 5.467 and 4.166 min, respectively. Standard curves were constructed by using these two columns. The obtained standard curves were Y = 136.7X + 8.1 (R^2^ = 0.9995, weight 1/x) and Y = 226.7X + 86.8 (R^2^ = 0.9739, weight 1/x), where X: concentration (ng/mL) and Y: peak area. Thus, a much better linear relationship was obtained by using the Accucore column. This is probably because this column is longer than the Zorbax column, which gave good separation from the interfering substances to decrease the matrix effect and achieve higher reproducibility. Therefore, the Accucore column was chosen for further study.

**Figure 2:**
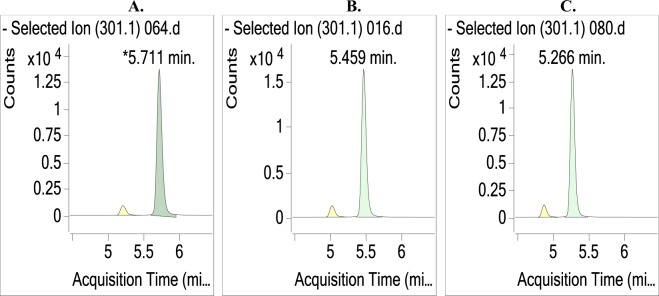
Effect of flow rate on the UHPLC-MS/MS chromatogram of the KA extract from *A. trifoliatus* leaves. (**A**) 0.3 mL/min, (**B**) 0.4 mL/min, (**C**) 0.45 mL/min. KA was separated on an Accucore C_18_ LC column and quantified with MRM transition at the quantitative fragment ion/product ion (*m/z*: 301.3/301.3) in ESI negative mode.

Three flow rates were tested for their method reproducibility, including 0.35, 0.4 and 0.45 mL/min. Three UHPLC-MS/MS chromatograms were obtained as shown in Fig. 2. From the figure, the KA peak shape was sharp and symmetrical with no tailing and very high resolution. The obtained analyte concentrations using the three flow rates were 4.0 ± 0.1, 4.0 ± 0.1, and 4.1 ± 0.2 μg/mg solid, and there were no significant differences in the test results between these three columns by the ANOVA *post-hoc* test. Therefore, from our results, 0.4 or 0.45 mL/min could be chosen as the flow rate, and we used 0.45 mL/min as the flow rate. The base peak width was reduced to 0.12 min when using 0.45 mL/min as the flow rate. By reducing the peak width, this could be utilized to enable faster analysis times while maintaining resolution.

**Figure 3:**
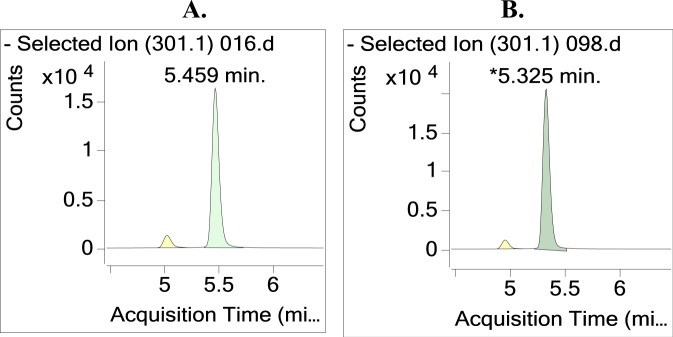
Effect of column temperature on the UHPLC-MS/MS chromatogram of the KA extract from *A. trifoliatus* leaves. (**A**) 35 °C, (**B**) 40 °C. KA was separated on an Accucore C_18_ LC column at a flow rate of 0.4 mL/min and quantified with MRM transition at the quantitative fragment ion/product ion (*m/z*: 301.3/301.3) in ESI negative mode.

Two different column temperatures were tested on method reproducibility, including 35 and 40 °C, and their corresponding UHPLC-MS/MS chromatograms are shown in Fig. 3. From the figure, both KA peak shapes are sharp and symmetrical with no tailing, and very high resolution, and there were no other interferences. The retention times from the two column temperatures were 5.46 and 5.32 min for 35 °C and 40 °C, respectively. The obtained analyte concentrations at the two temperatures were 4.0 ± 0.1 and 4.5 ± 0.5 μg/mg solid, and there was no significant difference between these two analyses. Therefore, in this study, we chose 35 °C as our column temperature.

**Figure 4:**
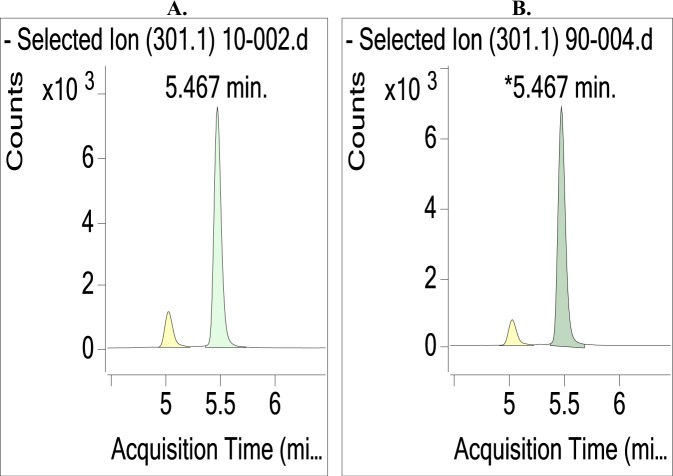
Effect of electrospray solvent on the UHPLC-MS/MS chromatogram of the KA extract from *Acanthopanax trifoliatus* leaves. (**A**) 2% FA in methanol; (**B**) 2% FA in 50% methanol. KA was separated on an Accucore C_18_ LC column at a flow rate of 0.4 mL/min and quantified with MRM transition at the quantitative fragment ion/product ion (*m/z*: 301.3/301.3) in ESI negative mode. UHPLC column temperature: 35 °C.

As indicated previously, electrospray solvent has a significant impact on the chromatogram and mass spectra of tandem mass spectrometry^25^. In this study, KA was extracted from *A. trifoliatus* leaves with 100% methanol. However, to improve the precision of the standard curve, 50% methanol was applied as a dilution solvent to prepare the standard solutions, and their corresponding UHPLC-MS/MS chromatograms are shown in Fig. 4. From the figure, the retention time from the two solvents was the same. The obtained concentration of analyte from the two solvents was also not significantly different.

In this study, we applied MRM as our MS/MS function. Very uniquely, we selected ion m/z 301.3 on Q1 by applying the appropriate Q2 (collision energy, 30 eV), and Q3 was obtained, but the product ion remained 301.3. This phenomenon indicated that KA lacked fragmentation under collision. Therefore, we chose the MRM transition as 301.3 > 301.3. These unique characteristics were also observed by another study^26^. Different collision energies were tested on KA from 0 eV to 40 eV and there was no unique fragments observed. Also the product ion with *m/z* 301.2 was the only ion detected when charging the collision energy from 0 to 40 eV on the Q2 which were shown in Supplement 2.

### Method validation

KA was detected in negative ion mode and was well-separated on the reversed phase column used. The KA molecule exhibited an adequately separate chromatographic peak that was easily distinguished from the baseline. Quantitation was performed based on retention time, which was compared with the authentic standard MRM transition.

Method selectivity was assessed by analyzing a blank, a positive sample and a KA standard solution. Selectivity met the criterion because there were no interfering peaks observed at the same retention times as the target analytes.

The calibration curve showed an acceptable linearity range and R^2^. For the equation Y = 136.7X + 8.1, X is in the range of 8 ng/mL~160 ng/mL, the weight is 1/*x*, and R^2^ = 0.9995. No unacceptable carryover was observed. Homoscedasticity should be tested in any linear regression analysis, especially for an analytical method in which the concentration range is broad^27^. By constructing the residual vs. concentration plot and applying the *F*-test, our calibration data did not meet the homoscedasticity requirement because the residuals are not randomly distributed. Thus, a weighted calibration curve should be constructed. Two different weighting factors (1/x and 1/x^2^) are compared, and the data are shown in Table 1. 1/x showed a smaller % RE than 1/x^2^, and thus, the 1/x weighting factor was chosen.

**Table 1:** Sum of the relative error (RE) of 3 replicates for KA at different standard concentrations.

Concentration (ng/mL)	No weighting	weighting	
		1/x	1/x^2^
8	259	110	197
16	110	86	95
40	74	40	51
80	10	13	15
160	15	14	17
Sum % RE	468	263	375

The LOQ was determined as 4 pg which was obtained by injection 0.5 μL of 8 ng/mL KA solution. At this concentration, signal to baseline noise *(S/N)* ratio was over 10 and bias (%) in accuracy and precision were below 20%. Lower concentration (6 ng/mL) was tested and found the precision was 35% and *S/N* < 10. Therefore LOQ was considered as the lowest calibration standard and be quantified reliably. The LOD was determined by *S/N* ratio of 3 and found LOD was 1.3 pg. These results were lower than those from an early report^26^.

Intra-day, inter-day and inter-person method repeatability were evaluated for 9 replicates. The obtained KA in the extract for the intra-day analysis by the same operator was 4.1 ± 0.1 μg/mg solid, and the R.S.D. was 2.8%. The obtained KA in the extract for the inter-day analysis by the same operator was 4.0 ± 0.1 μg/mg solid, and the R.S.D. was 3.2%. The values of KA concentration from operator 1 and operator 2 were 4.0 ± 0.1 μg/mg solid and 4.2 ± 0.1 μg/mg solid, respectively. The R.S.D. of inter-person was 3.6%. The KA in the extract obtained by different instruments of the same model (Agilent 6460) on the same day was 4.1 ± 0.1 μg/mg solid, and the R.S.D. was 2.9%. Therefore, all precision values were below 15%, which met the EMA guideline requirements^28^.

Accuracy was expressed as the recovery, determined by analyzing 3 replicates of three quality controls (QCs) spiked in the sample for extract. The three quality controls (QCs) were prepared from the KA standard stock solutions corresponding to 3 points, a low QC, medium QC and high QC. The final concentrations of three QCs were 1.81, 3.62 and 6.03 μg/mg solid. The recoveries (%) were 99.2 ± 8.0, 99.7 ± 2.7 and 97 ± 5.9. The detailed results about accuracy test were shown in Supplement 3. This shows that the method met the requirements of the EMA guidelines and that the recovery was in the range of 75~110%^28^. A minor matrix effect of 94% was observed with the extract as the matrix. MAX SPE purification significantly decreased the matrix effect. The accuracy and matrix effect results were shown in Table 2.

**Table 2:** Accuracy and matrix effect for KA at different standard concentrations.

QC concentration (μg/mL)	Matrix effect (%)	Intra-day accuracy (%)
1.81	95.2 (1.2)	99.2 (8.0)
3.62	93.8 (2.4)	99.7 (2.7)
6.03	95.4 (3.2)	97.0 (5.9)

Standard deviation was given in the parentheses.

## Conclusions

In this study, a UHPLC-MS/MS method was developed as a new assay procedure and was evaluated and applied for specific quantitation of KA content in *A. trifoliatus* leaves after purification by a SPE cartridge. After the implementation of an ultrasound-assisted extraction, this UHPLC-MS/MS method was validated in-house with the plant extract demonstrating good recovery rates above 97% and good R.S.D. This method showed very good selectivity and sensitivity with a LOD of 1.3 pg and a LOQ of 4 pg. The method has been applied successfully in the determination of KA content in *A. trifoliatus* leaves, which can reach 0.4% (4 μg/mg solid).

## Methods

### Chemicals

LC-MS grade water (LC-MS, LiChrosolv), acetonitrile (LC-MS, LiChrosolv), methanol (LC-MS, LiChrosolv) and formic acid (reagent grade, 95%) were obtained from Sigma-Aldrich (Shanghai, China). The KA standard with a purity over 98% was obtained from the National Drug Reference Standards Center (Beijing, China). *A. trifoliatus* leaves were picked in April 2018 from a farm in Enping, Guangdong Province and shipped to Jinan University on the same day under refrigerated conditions. Upon arriving at the lab, the fresh leaves of *A. trifoliatus* were washed, dried in an air flow, ground in liquid nitrogen, lyophilized for 2 days and then powdered with an electronic mill (20-mesh sieve). The powder was kept in centrifuge tubes sealed with parafilm and reserved in dry and cool conditions until analysis.

### Extraction KA from *A. trifoliatus*

The extraction procedure of KA from *A. trifoliatus* Merr. was optimized for plant leaf powder in terms of sample mass and ultrasound-assisted extraction time. A set of three replicates of plant leaf powder were weighed into a volumetric flask with a cap, and 100 mL methanol was added as the extraction solvent. The mixture was first vortexed and then sonicated for a certain amount of time, including 15, 30 and 60 min. An ultrasonic water bath (Powder sonic 420, with a 40 kHz frequency, maximum power of 700 W, and internal dimensions (id) of 500 × 300 × 150 mm) was utilized for this purpose. After sonication, the mixture was cooled to room temperature and then passed through vacuum filter paper (Whatman qualitative filter paper, Grade 1, Sigma-Aldrich), and the supernatant was recovered. Different amounts of powder were tested for extraction, including 0.1, 0.2, 0.3 and 0.5 g. Ultrasound-assisted extraction times included 15, 30 and 60 min. The 5 mL of extract was evaporated completely using a vacuum evaporator centrifuge (Labconco, Fisher Scientific, USA), dissolved in 10 mL of water and cleaned up by solid phase extraction (SPE).

### SPE clean up

The KA extract was further cleaned up by an Oasis MAX (mixed-mode anion exchange, 60 mg, 3 cc, Waters) cartridge column for acidic compounds. The Oasis MAX cartridge was first activated and conditioned with 10 mL of methanol and 10 mL of Mill-Q water. Then, 0.25 mL of the extract was applied to the cartridge. Next, the column was washed with 10 mL of 5% NH_4_OH in water and dried for 5 min under a stream of air. The analytes were then eluted with 10 mL of 2% FA in methanol and further filtered through a 0.22 μm nylon filter before injection into the UHPLC-MS/MS. The extract was diluted 80-fold to obtain an extract concentration in the range of the linear standard curve.

### UHPLC-MS/MS conditions

Chromatographic separation of KA from the other matrix components was carried out by reversed phase chromatography on an Agilent 1290 Infinity UHPLC equipped with a binary pump, autosampler, column oven and degasser (Agilent Corp., Santa Clara, CA). The mobile phase was constituted as follows: 0.1% FA in water (solution A) and acetonitrile (solution B). For the determination of KA, different chromatographic columns were tested in this study, including an Accucore C_18_ LC column (100 × 2.1 mm, 2.6 μm, Thermo Scientific, USA) and a Zorbax Eclipse Plus C_18_ column (2.1 × 50 mm, 1.8 μm, Agilent Technologies, USA). Different flow rates for KA recovery and method precision were tested, including 0.35, 0.4 and 0.45 mL/min. KA chromatographic separation was performed using the following gradient elution: 0–1 min, 90% A; 1–3 min, 90% A to 60% A; 3–4 min, 60% A-10% A; 4–6.5 min, 10%, then return to the initial conditions and equilibrate the column for 1 min. The injection volume was 0.5 μL, the autosampler was kept at 20 °C and column temperature was investigated at 35 °C and 40 °C.

The UHPLC eluate was interfaced with an Agilent 6460 triple quadrupole mass spectrometer (Agilent Corp.) operating in negative ion electrospray mode. An Agilent Mass Hunter workstation was used for control of the equipment, data acquisition and analysis. For optimization of the MS/MS parameters, a KA standard solution prepared in methanol was infused into the mobile phase (0.5 mg/mL) at a flow rate of 20 μL/min using a syringe pump (Harvard Apparatus, Holliston, MA) to tune the instrument. Nitrogen was used both as the drying gas at a flow rate of 11 L/min and as the nebulizing gas at a pressure of 45 psi. The drying gas temperature was 300 °C, and a potential of 4 kV was applied across the capillary. Finally, the instrument was operated with a fragmentor voltage of 140 V. Acquisition was performed by MRM, wherein the respective pseudomolecular anion of KA was fragmented by collision-induced dissociation into a fragment for detection. The MRM transition and the collision voltage were optimized, and the value of the collision energy for the qualitative and quantitative ion (301.1) was 30 eV. The dwell time was 100 ms.

### Method validation

The developed method was validated based on the EMA guidelines, including selectivity, carryover, limit of detection and limit of quantification, linearity, repeatability, precision, accuracy and matrix effect^28^.

#### Calibration curves and linearity

(1) KA stock solutions were prepared by fully dissolving 1 mg of KA into a 10 mL brown volumetric flask in methanol. The concentration of the stock solution was 0.1 mg/mL. The stock solution was completely mixed and stored at −80 °C. (2) KA intermediate standard solutions were prepared by pipetting 1 mL of the stock solution into a 25 mL brown volumetric flask and bringing up to volume with 50% methanol. The final concentration of the intermediate standard solution was 4 μg/mL. The intermediate standard solutions were also stored at −80 °C. (3) The working solutions of KA were prepared by pipetting 20 μL, 40 μL, 100 μL, 200 μL and 400 μL of the intermediate solutions into a 10 mL brown volumetric flask and bringing up to volume with 50% methanol. The final concentrations of the working solutions were 8, 16, 40, 80 and 160 ng/mL. The ranges of the calibration curves were determined from the preliminary analysis of KA in *A. trifoliatus* leaves. A 1/*x* weighted linear regression was applied so that the back-calculated concentration should be within 15% of the theoretical concentration.

#### Solvent effects in tandem mass spectrometry

An early study discovered that chromatograms and mass spectra may not only be dependent on careful control of instrumental parameters such as tuning conditions, but also on the electrospray solvent^25^. In this study, KA was extracted from *A. trifoliatus* leaves with methanol as the extraction solvent. However, to improve the precision of the standard curve, 50% methanol was applied as the dilution solvent to prepare the standard solutions. Therefore, the chromatograms of KA in methanol and 50% methanol were compared in terms of peak shape, peak area and retention time.

The LOQ was considered as the lowest concentration of analytes that can be quantified reliably so that the bias (%) in accuracy and precision were below 20%. A signal-to-ratio of 3 and 10 is used for estimating LOD and LOQ, respectively. The LOQ and LOD were determined by diluting the KA standard solution until the *S/N* ratio reached to 10 and 3, respectively.

#### Selectivity and carryover

The selectivity refers to the method that can differentiate the target analyte from other matrix components. This was assessed by analyzing negative samples, positive samples and KA standard solutions. The presence of possible endogenous or exogenous interferences was verified by monitoring the MRM chromatogram. The MRM chromatogram was specific for KA at its expected retention time. The acceptance criterion for the selectivity was that the peak area of interference should be below 20% of the peak area of the LOQ of KA.

Carryover was evaluated by injecting blank samples after the run of the highest calibration concentration. The acceptance criteria for carryover were the same as for the evaluation of selectivity.

#### Robustness

To assess the robustness of the method, 9 replicates of the sample extraction were prepared by the same operator under the same instrument on the same day.

#### Precision

The precision was evaluated by the precision of 2 different operators on 3 nonconsecutive days, and by two instruments of the same model with 9 replicates of sample extraction.

#### Accuracy

The accuracy was expressed as the recoveries, determined by analyzing 3 replicates of three quality controls (QCs) spiked in the sample for extract. Three quality controls (QCs) were prepared from the KA standard stock solutions with 3 points, low QC, medium QC and high QC. The final three QCs were 1.81, 3.62 and 6.03 μg/mg solid, which corresponded to 0.5-, 1- and 1.5-fold the KA concentration in the plant extract. 0.5 mL individual QC stock solution (362, 724 and 1206 μg/mL) was spiked in the same amounts of samples and gone through ultrasound-assisted extraction, concentration and SPE clean-up. The final extracts were determined in the same day. The accuracy (recovery) was calculated with the following equations:

I $${\rm{Accuracy}}\,( \% )=\frac{({A}-{B})\ast 100}{{C}}$$

A: KA concentration in sample extracts spiked with individual QC standard solution; B: KA concentration in sample extracts; C: nominal value of KA in QC standard solution. The KA concentration obtained from A and B were calculated against the calibration curve.

#### Matrix effect

The matrix effect was also determined for the method. The sample matrix, such as coeluting compounds, can contribute to alterations in the analyte ionization and overall response. Therefore, complete separation between the analyte and the coeluting matrix could help to decrease or increase the ionization of the target analyte. The matrix effect was determined with the following method. The volume of 10 μL QCs (1.81, 3.62 and 6.03 μg/mL) were spiked into 1 mL post sample extract. The peak area obtained in the matrix was compared with the corresponding peak area in the extraction solution with the same concentration. Each sample was analyzed three times. The matrix effect was calculated as follows:

II $${Matrix}\,{effect}\,( \% )=({A}-{B})\times 100/{C}$$

A: mean peak area in the matrix after spiking the QCs; B: endogenous KA peak area in the matrix before spiking the QCs; and C: mean peak area of three QCs in the extraction solution.

## Supplementary information

Supplementary information.
