# Supplementary material for: Determination of Kaurenoic Acid in Acanthopanax trifoliatus by Ultra-High Performance Liquid Chromatography Coupled with Tandem Mass Spectrometry (UHPLC-MS/MS)
Source: Sci Rep. 2020 Feb 25;10:3378. doi: 10.1038/s41598-020-60426-3 (PMC7042316; doi:10.1038/s41598-020-60426-3)
Supplement: Supplementary file 1 — Supplementary information. [file 41598_2020_60426_MOESM1_ESM.docx]

**Determination of Kaurenoic Acid in *Acanthopanax trifoliatus* by Ultra-High Performance Liquid Chromatography Coupled with Tandem Mass Spectrometry (UHPLC-MS/MS)**
**Qun Peng^1^, Jianyuan Chen^2^, Hanying Duan^1*^, Chao Wang^1^***
*1. Department of Food Science and Technology, Jinan University, Guangzhou, 510632, China*
*2. Division of Research and Development, Kingmed Diagnostics, Guangzhou, 510330, China*

**Supplement 1** Different solid extraction amounts on the content of KA

| solid weighting (g) | Final volume (mL) | KA determined concentration (μg/mL) | KA content in solid (μg/mg solid) |
| --- | --- | --- | --- |
| 0.13±0.02 | 100 | 5.01±0.78 | 4.02±0.06 |
| 0.21±0.00 | 100 | 8.29±0.07 | 4.09±0.13 |
| 0.31±0.00 | 100 | 12.73±0.24 | 4.23±0.11 |
| 0.51±0.01 | 100 | 20.65±0.26 | 4.08±0.10 |

**Supplement 2**

collision energy: 0 eV

collision energy: 0 eV

collision energy: 10 eV

collision energy: 10 eV

collision energy: 20 eV

collision energy: 20 eV

collision energy: 30 eV

collision energy: 30 eV

collision energy: 40 eV

collision energy: 40 eV

**Supplement 3**

Accuracy (recovery) test results

| Samples (g) | QC concentration (μg/mg solid) | KA in extract (μg/ mg solid) | KA in extract with QC (μg/ mg solid) | Intra-day accuracy (%) |
| --- | --- | --- | --- | --- |
|  |  |  |  |  |
| 0.136 0 | 1.81 | 4.96 | 6.92 | 108.2 |
| 0.114 7 |  | 4.02 | 5.70 | 92.7 |
| 0.121 1 |  | 4.23 | 5.98 | 96.7 |
| 0.118 0 | 3.62 | 4.04 | 7.62 | 98.8 |
| 0.120 5 |  | 4.13 | 7.66 | 97.6 |
| 0.132 3 |  | 4.91 | 8.63 | 102.8 |
| 0.101 2 | 6.03 | 3.61 | 9.56 | 98.6 |
| 0.104 7 |  | 3.74 | 9.88 | 101.9 |
| 0.107 8 |  | 3.85 | 9.31 | 90.5 |
